# Isolated colorectal liver metastases locally progressing after stereotactic body radiotherapy rescued with surgery

**DOI:** 10.3747/co.v16i5.360

**Published:** 2009-09

**Authors:** J.M. Gasent Blesa, J. Laforga Canales, A. Alberola Soler, F. Peiró Monzó, J. Bertelli Puche, V. Alberola Candel

**Affiliations:** * Hospital General Universitari Marina Alta, Dènia, Alacant, Spain; † Surgical Department, Hospital General Universitari Marina Alta, Dènia, Alacant, Spain; ‡ Hospital Universitari Arnau de Vilanova, València, Spain

**Keywords:** Liver metastases, colon cancer, liver radiosurgery, salvage surgery

## Abstract

Treatment of patients with metastatic colorectal cancer has changed in recent years, with many patients now being offered intent-to-treat regimens. In this context, a multidisciplinary approach to the metastatic disease may lead to individualized treatment for any patient. Stereotactic body radiotherapy (sbrt) is not the most common treatment. Here, we present the clinical case of a patient with a solitary liver metastasis initially treated with sbrt that was rescued with surgery when a local recurrence was detected.

## 1. INTRODUCTION

Nearly 150,000 Americans are diagnosed with colorectal cancer (crc) annually, with one third dying from their disease, most from metastatic tumours 1. Liver is the main metastatic site for patients with crc, and although two thirds of affected patients experience extrahepatic spread, in some cases the disease is isolated to the liver. For patients with isolated liver metastases, regional treatment approaches may be considered as an alternative to systemic chemotherapy.

The available regional treatments for hepatic metastases from crc include surgical resection, local tumour ablation (that is, instillation of alcohol or acetic acid directly into the metastatic lesions), cryotherapy, radiofrequency ablation, regional hepatic intra-arterial chemotherapy or chemoembolization, and radiation therapy.

Although hepatic resection was once reserved for patients with a maximum of three lesions in the same lobe (if achieving 1-cm margins was possible) and without portal lymph node metastases, all of these “rules” have been challenged today, particularly with advances in surgical technique and more effective systemic therapies 2. As a result, there are no widely accepted criteria defining the patients that are best suited for surgical therapy, and most clinicians take an aggressive stance in the management of hepatic metastases.

Local tumour ablation can be accomplished with direct intratumoral instillation of alcohol 3 or acetic acid 4 or with hyperthermic ablation 5 or cryotherapy 6. In general, lesions that are amenable to surgical resection also lend themselves to ablative treatments. Thus, local ablative methods may be considered a less morbid alternative to surgical resection in patients who are at high risk or are otherwise not candidates for surgery 7.

Since the late 1990s, technological advances in radiation planning [conformal radiation therapy, intensity-modulated radiation therapy, stereotactic body radiation therapy (sbrt)] and delivery (image-guided radiation therapy, breathing motion management strategies) have allowed high-dose external-beam radiation therapy to be safely delivered focally to liver metastases, extending the role of radiation therapy to definitive therapy, with the potential for eradication of the disease and possibly cure in appropriately selected patients. In addition, radiation therapy may play a role in the downstaging of border-line resectable tumours after failure of chemotherapy to achieve this goal, when used in conjunction with other liver-directed therapies.

### 1.1 SBRT

The use of sbrt, which refers to a limited number of high-dose fractions delivered very conformally to extracranial targets using radiation doses higher than those used in standard fractionation, in the treatment of liver metastases is not common, but has been established 8–12. With sbrt, highly conformal radiation therapy can be delivered in far fewer treatments than are possible with conventional radiation therapy. The shorter treatment times with sbrt can overcome tumour repopulation, and the technique is therefore advantageous in terms of resource utilization and patient convenience. Liver toxicity following sbrt has seldom been observed, largely because most of the tumours being treated are small (predominantly less than 8 cm in their maximum dimension), usually requiring less than 25% of residual liver to be irradiated.

Safety for sbrt in 1–10 fractions has been described in several retrospective series and more recently confirmed in prospective dose escalation studies. The Swedish group led by Blomgren^8^ was the first to use sbrt to treat liver metastases. In 1995, they reported a response rate of 43% for 14 liver metastases treated with 20–45 Gy in 1–4 fractions, with a prolonged time to maximum response (approximately 16 months for a 13-cm liver metastasis). No liver toxicity was observed, but 1 patient developed grade 4 hemorrhagic gastritis. In a 1998 update 13, the local control rate was 95% with a mean survival of 17.8 months after sbrt for 21 patients with liver metastases. In another series 14, 20 Gy in 2 fractions or 15 Gy in 3 fractions was used, with no serious toxicity in patients with recurrent liver metastases following resection. The subsequent local control ranged between 13 months and 101 months.

A group from Germany 9 used sbrt in a 3-fraction trial. They recruited 51 patients with liver metastases; of these patients, 23 had crc liver metastases. These authors found reduced local control for crc metastases as compared with metastases from other cancer types, as others have also observed. That finding may be a result of the rationale of treating crc liver metastases with ablation or surgery, which may in turn influence patient selection, so that patients referred for sbrt are later in their natural history.

The maximum tolerated dose to liver using sbrt was not reached in a prospective study of 14–26 Gy in 1 fraction in 60 liver tumours (56 metastases). Median tumour volume was 10 mL (range: 1–132 mL). Local control was 81% at 18 months 15. A North American prospective study of 18 patients with 25 tumours of no more than 6 cm demonstrated the feasibility of 3 fractions of sbrt up to 20 Gy 16.

A Canadian sbrt study described the safety of delivering sbrt in 6 fractions, using an individualized dose allocation in liver cancers ranging from 3 ml to 3000 ml 17. This phase i/ii study was conducted by Dawson *et al.* at Princess Margaret Hospital in Toronto. Their work encompassed sbrt with individualization of immobilization, radiation planning, margin determination for the planning target volume, image guidance strategy, and prescription dose. Breath-hold was used to immobilize the liver when feasible. Image-guidance strategies included orthogonal megavoltage images and orthogonal kilovoltage fluoroscopy using the diaphragm as a surrogate for the liver, and kilovoltage cone-beam computed tomography (ct) using the liver or the tumour for guidance. The prescription dose was individualized to maintain the same estimated risk of radiation-induced liver disease, based on a normal-tissue complication probability model, with a maximum permitted dose of 60 Gy in 6 fractions.

### 1.2 Patient Selection for SBRT

Ideally, sbrt should be used for patients with no more than 5 focal metastases less than 8 cm maximum in diameter (because local control is better for smaller tumours), not adjacent to the stomach or the small bowel, with a breathing motion of less than 5 mm. Anatomically, patients with tumours distant from gallbladder, caudate lobe, and capsule are more suitable. More commonly, patients may have less liver volume, more breathing motion (up to 30 mm), and more lesions. As the tumour factors reduce the benefit of local control and as risk of toxicity increases, the rationale for radiation therapy is reduced.

The most challenging patients to treat are those with underlying liver disease such as cirrhosis or hepatitis (viral hepatitis must be treated before radiation is delivered), with less than 700 mL of uninvolved liver, with more than 5 metastases, and with a larger respiratory motion (>30 mm). Tumours close to the stomach and duodenum are the most challenging and difficult to cure, because stomach and duodenum tolerances are below the doses most likely to control liver metastases.

## 2. CASE REPORT

A 67-year-old man with previous history of rectosigmoid adenocarcinoma and rising serum carcinogenic embryonic antigen (cea), presented to our hospital for a second opinion.

This patient’s history revealed the diagnosis of rectosigmoid adenocarcinoma cT3N0M0 at another centre in November 2004. He was treated with neoadjuvant chemoradiation therapy, receiving oral capecitabine 835 mg/m^2^ twice daily until January 2005 and completing 50.4 Gy radiation therapy with good tolerance.

Surgery was performed in February 2005. The pathology from a low anterior resection confirmed a low-grade adenocarcinoma that had infiltrated the surrounding fat, with 3 positive lymph nodes from a total of 10 isolated.

The patient then received 6 cycles of adjuvant chemotherapy with capecitabine 1250 mg/m^2^ on days 1–14 every 21 days, without any relevant toxicity.

In July 2005, ct and magnetic resonance imaging of the liver resulted in a diagnosis of liver progression with a solitary liver metastasis to the viii segment and rising serum cea (to 34.5 ng/mL). The patient then began chemotherapy with folfox6–bevacizumab every 4 days for a total of 18 cycles. The response to this regimen was considered a partial response, and the patient was evaluated for surgical resection of the metastasis. However, because of the proximity of the vascular axis of the liver, he was deemed not amenable for resection and was recommended for sbrt.

In May 2006, he was treated with liver sbrt in 4 sessions, for a total of 36.5 Gy. A subsequent control positron-emission tomography (pet) scan was negative, and the patient was considered to be in complete clinical response. He then continued chemotherapy with oral capecitabine 1250 mg/m^2^ on days 1–14 every 21 days, until the visit to our clinic. Before this consultation, his serum cea had risen to 12 ng/ml from 4 ng/mL at the time of his first evaluation, and no change in the treatment plan had been discussed. A pet–ct was then requested.

**FIGURE 1:**
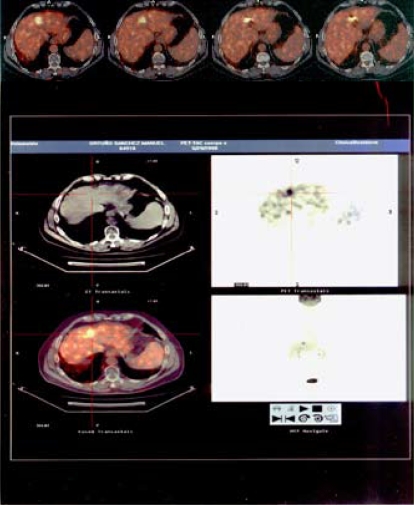
A metabolic lesion with a standardized uptake value of 18.7 was found at the hepatic viii segment.

The pet–ct (Figure 1) showed activity at the viii liver segment, with a standardized uptake value of 18.7 for the same area in which the tumour had previously progressed and been treated with radiosurgery.

The case was discussed at our tumour board, and we decided to offer metastasis resection.

**FIGURE 2:**
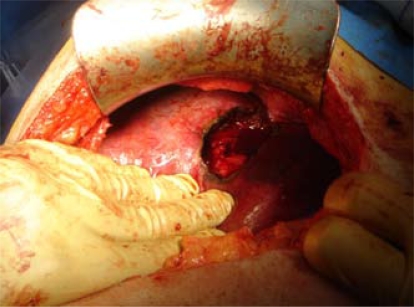
Resection of the liver lesion.

After a full colonoscopy, which revealed no lesions, the patient was operated on, and most of the lesion was resected (Figure 2). Examination of the abdominal cavity showed no abnormality, and some biopsies were taken.

**FIGURE 3:**
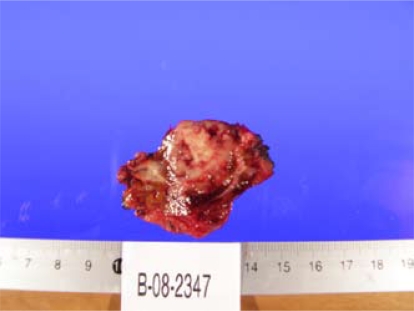
Hepatic cut section of a well-delineated tumour mass measuring 2.5×2×2 cm, whitish and firm.

**FIGURE 4:**
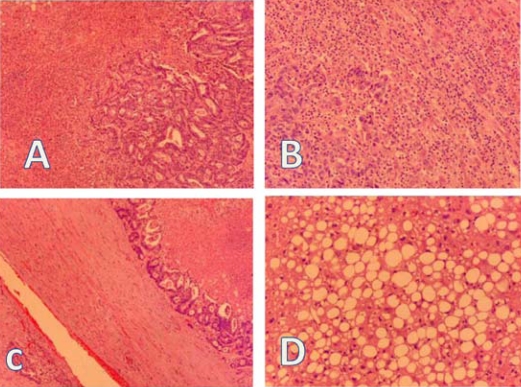
(A) Metastatic colonic adenocarcinoma showing a tubular growth pattern. (B) Focally, the tumour showed heavy lymphocytic infiltration and small-vessel proliferation. (C) Metastatic adenocarcinoma infiltrating the adventitia of a large venule. Note the garland necrosis. (D) Surrounding hepatic tissue showing macrovesicular steatosis. All panels: Hematoxylin and eosin stain, 400× magnification.

A hepatic biopsy was submitted for diagnosis. Grossly, the specimen measured 6.5×5×3 cm (Figure 3). The capsule was smooth. At cut section, a well-delineated tumour mass measuring 2.5×2×2 cm, whitish and firm, was observed. Microscopically (Figure 4), the tumour consisted of large atypical glands, with a tubular and cribriform pattern of growth and garland dirty necrosis in the centre. The tumour cells exhibited nuclear pleomorphism. Focally, the interface of the tumour and the hepatic tissue showed heavy inflammatory infiltration with microvascular proliferation. The tumour infiltrated the adventitia of a large venule. The surrounding hepatic tissue showed macrovesicular steatosis and narrowing of the small terminal hepatic veins with loose subintimal mesenchyme and fibrosis. The surgical margins were free of tumour.

No complication was recorded during the post-operative period, and the patient was discharged on the third day following surgery.

## 3. DISCUSSION

The main potentially curative option for patients with an isolated liver crc metastasis is surgical resection. For appropriately selected patients with 4 or fewer metastases, the 5-year relapse-free survival rate averages 30%; in at least four contemporary series, the 5-year overall survival rate is approximately 58% 18,19.

New techniques such as radiofrequency ablation or sbrt may offer extended survival for selected patients and warrant the inclusion of sbrt as part of an intent-to-treat strategy. Fewer than 20% of patients may experience local recurrence 8,20,21, and even if selection bias is believed to exist in the series data, the results of sbrt remain impressive. Our case also reflects the role played by the new imaging techniques and the importance of cea in managing crc.

## 4. CONCLUSIONS

The case presented here is an example of the necessity for managing metastatic crc in multidisciplinary groups in which all of the therapeutic options are discussed. Integration of radiotherapists into the multidisciplinary management of patients with metastatic crc is, in our opinion, mandatory.
